# Seroma formation after surgery for breast cancer

**DOI:** 10.1186/1477-7819-2-44

**Published:** 2004-12-09

**Authors:** Esmat Hashemi, Ahmad Kaviani, Masoume Najafi, Mandana Ebrahimi, Homeira Hooshmand, Ali Montazeri

**Affiliations:** 1 Iranian Center for Breast Cancer, Tehran, Iran; 2 Tehran University of Medical Sciences, Faculty of Medicine, Department of Surgery, Tehran, Iran

## Abstract

**Background:**

Seroma formation is the most frequent postoperative complication after breast cancer surgery. We carried out a study to investigate the effect of various demographic, clinical and therapeutic variables on seroma formation.

**Patients and methods:**

A retrospective cross sectional study of patients who underwent surgical therapy for breast cancer with either modified radical mastectomy (MRM) or breast preservation (BP) was carried out. The demographic data and clinical information were extracted from case records. Seroma formation was studied in relation to age, type of surgery, tumor size, nodal involvement, preoperative chemotherapy, surgical instrument (electrocautery or scalpel), use of pressure garment, and duration of drainage. The multiple logistic regression analysis was performed to estimate odds ratios.

**Results:**

A total of 158 patients with breast cancer were studied. The mean age of the patients was 46.3 years (SD ± 11.9). Seventy-three percent underwent modified radical mastectomy and the remaining 27% received breast preservation surgery. Seroma occurred in 35% of patients. In multivariate logistic regression analysis an association of postoperative seroma formation was noted with modified radical mastectomy (OR = 2.83, 95% CI 1.01–7.90, P = 0.04). No other factor studied was found to significantly effect the seroma formation after breast cancer surgery.

**Conclusion:**

The findings suggest that the type of surgery is a predicting factor for seroma formation in breast cancer patients.

## Background

Breast cancer is the second leading cause of cancer death among women. The surgical treatment of choice for these patients is either modified radical mastectomy or breast preservation depending upon stage of the disease. Seroma formation is the most frequent postoperative complication after breast cancer surgery. It occurs in most patients after mastectomy and is now increasingly being considered side effect of surgery rather than a complication however, all patients are not clinically symptomatic [1]. Seroma is defined as a serous fluid collection that develops under the skin flaps during mastectomy or in the axillary dead space after axillary dissection [2]. Incidence of seroma formation after breast surgery varies between 2.5% and 51% [3-5]. Although seroma is not life threatening, it can lead to significant morbidity (e.g. flap necrosis, wound dehiscence, predisposes to sepsis, prolonged recovery period, multiple physician visits) and may delay adjuvant therapy [6,7]. Fluid collection is ideally managed by repeated needle aspiration to seal the skin flaps against the chest wall. Several factors have been investigated as the cause of seroma formation these include age, duration of wound drainage, use of pressure garment, postoperative arm activity, preoperative chemotherapy, and use of electrocautery [3,8-12]. The present study was undertaken to identify risk-factors associated with seroma formation after breast cancer surgery.

## Patients and methods

A cross sectional study of a consecutive sample of 158 patients attending the breast cancer clinic between January 2000 to October 2002 in Tehran, Iran, was carried out. All patients undergoing surgical therapy [modified radical mastectomy (MRM) or breast preservation (BP)] were included. Level II axillary lymph node dissection was performed for both groups. None of the patients underwent immediate reconstruction. The demographic data and clinical information were extracted from case records. Axillary seroma was defined as any clinically apparent fluid collection in the axilla or under the skin flaps and was treated with multiple needle aspirations. Seroma formation was studied in relation to age, type of surgery, tumor size, nodal involvement, preoperative chemotherapy, surgical instrument (electrocautery or scalpel), use of pressure garment, and duration of drainage. To analyze data univariate odds ratio (or relative risk) was calculated using Chi-square tests or regression analysis and this was followed by the multivariate logistic regression analysis to evaluate independent risk factors related to seroma formation. The variables of interest were selected in a single step (enter method), classification cut off was  set at 0.5, probably of step for entry into the model was set at 0.05 and  removal at 0.1, and the model was set to converge in maximum of 20  iterations. All variables under study were considered as independent predicting factors and seroma formation was considered as dependent variable for multivariate analysis. The study was approved by the institutional ethics committee.

## Results

In all, 158 breast cancer patients were recruited into the study and 55 patients developed seroma, giving an overall incidence of 35% for seroma formation after breast surgery. The mean age of patients was 46.3 years (SD ± 11.9). One hundred and fifteen patients (73%) underwent MRM and BP was performed in 43 patients (27%). The axillary node involvement was significantly different between MRM and BP patients (χ^2 ^= 4.52, df = 1, P = 0.03) indicating that those who underwent MRM had higher rate of positive axillary nodes compared to those who received BP (78% vs. 21% respectively). Thirty-one mastectomies were performed by scalpel dissection of the skin flap (20%) and 127 by cautery dissection (80%). Two closed suction drains were placed in all patients undergoing surgery. Sixty-six percent of patients (n = 104) were node positive and the remaining 34% (n = 54) were node negative. The patients' characteristics and univariate odds ratios are shown in Table 1.

**Table 1:** The characteristics of patients in seroma and no seroma groups and univariate odds ratio.

	**Seroma group (n = 55) n. (%)**	**No seroma group (n = 103) n. (%)**	OR (95% CI)*	p value**
***Age (years)***				*0.22*
<40	12 (21.8)	34 (33.0)	1.00 (ref.)	
40–49	20 (36.4)	38 (36.9)	1.49 (0.63–3.49)	
>50	23 (41.8)	31 (30.1)	2.10 (0.89–4.92)	
***Tumor size (cm)***				*0.64*
<2	21 (38.2)	47 (45.6)	1.00 (ref.)	
2–5	21 (38.2)	34 (33.0)	1.42 (0.67–3.01)	
>5	13 (23.6)	22 (21.3)	1.26 (0.53–2.96)	
***Nodal involvement (n = 152)***				*0.31*
No	14 (26.4)	34 (36.8)	1.00 (ref.)	
Yes	39 (73.6)	65 (65.7)	1.45 (0.69–3.04)	
***Surgical procedure***				*0.03*
Breast conservation	10 (18.2)	33 (32.0)	1.00 (ref.)	
Modified radical mastectomy	45 (81.8)	70 (68.0)	2.12 (0.95–4.72)	
***Surgical instrument***				*0.06*
Scalpel	8 (14.5)	23 (22.3)	1.00 (ref.)	
Cautery	47 (85.5)	80 (77.7)	1.68 (0.70–4.07)	
***Neoadjuvant chemotherapy***				*0.22*
No	46 (83.6)	93 (90.3)	1.00 (ref.)	
Yes	9 (16.4)	10 (9.7)	1.82 (0.69–4.78)	
***Pressure garment***				*0.63*
Yes	12 (21.8)	26 (25.2)	1.00 (ref.)	
No	43 (78.2)	77 (74.8)	1.21 (0.55–2.63)	
***Axillary drainage time (days/n= 152)***				*0.64*
>10	8 (15.1)	20 (20.2)	1.00 (ref.)	
5–10	30 (56.6)	49 (49.5)	1.53 (0.59–3.90)	
<5	15 (28.3)	30 (30.3)	1.25 (0.44–3.49)	

* Odds ratios derived from univariate logistic regression analysis.

** P values derived from the Chi-squared test.

The results of  multivariate logistic regression analysis indicated that only the surgical type was significantly associated with seroma formation (OR = 2.83, 95% CI 1.01–7.90, P = 0.04). Of patients with BP, 10 of 43 (23%) developed seroma, while those who underwent MRM 45 of 115 (39%) developed seroma. The seroma formation did not show any significant association with any other variables studied. The results of maultivariate analysis are shown in Table 2.

**Table 2:** Risk factors for seroma formation derived from the multivariate logistic regression analysis

	**β (SE)**	**Wald**	**P**	**OR (95% CI)**
***Age (years)***				
<40	-	-		1.00 (ref.)
40–49	0.68 (0.49)	1.92	0.16	1.99 (0.75–5.25)
>50	0.75 (0.48)	2.44	0.11	2.12 (0.82–5.44)
***Tumor size (cm)***				
<2	-	-		1.00 (ref.)
2–5	0.38 (0.44)	0.74	0.38	1.46 (0.61–3.49)
>5	0.08 (0.52)	0.03	0.88	1.09 (0.39–3.00)
***Nodal involvement (n = 152)***				
No	-	-		1.00 (ref.)
Yes	0.35 (0.43)	0.66	0.41	1.42 (0.60–3.35)
***Surgical procedure***				
Breast conservation	-	-		1.00 (ref.)
Modified radical mastectomy	1.04 (0.52)	3.97	0.04	2.83 (1.01–7.90)
***Surgical instrument***				
Scalpel	-	-		1.00 (ref.)
Cautery	0.60 (0.51)	1.36	0.24	1.83 (0.66–5.07)
***Neoadjuvant chemotherapy***				
No	-	-		1.00 (ref.)
Yes	0.33 (0.55)	0.37	0.54	1.40 (0.47–4.13)
***Pressure garment***				
Yes	-	-		1.00 (ref.)
No	0.51 90.46)	1.28	0.26	1.67 (0.67–4.11)
***Axillary drainage time (days/n= 152)***				
>10	-	-		1.00 (ref.)
5–10	0.13 (0.59)	0.04	0.76	1.17 (0.41–3.32)
<5	0.16 (0.53)	0.09	0.82	1.14 (0.35–3.66)

## Discussion

Breast cancer is the most common malignancy in women. Surgery is the mainstay of treatment. Modified radical mastectomy with or without reconstruction or breast preservation in addition to axillary lymph node dissection are common surgical procedures in breast cancer. Surgery of the axilla is associated with numerous complications, including infection, lymphedema of the ipsilateral upper extremity and collection of fluid in surgical site (seroma). Most common complication after breast cancer surgery is wound seroma. The exact etiology of seroma formation remains controversial. Several interventions have been reported with the aim of reducing seroma formation including the use of ultrasound scissors in performing lymphadenectomy [13], buttress suture [14], fibrin glue [15], fibrin sealant [16], bovine thrombin application [17], and altering surgical technique to close dead space [18]. However, it has been suggested that although the use of these interventions might reduce the risk of seroma formation, further studies are needed to verify the real impact on long-term morbidity of such techniques [19].

Several studies have been performed to investigate factors related to post-surgical seroma. These studies have observed that the early removal of drains might led to increased incidence of seroma [12], whereas others have shown that drains removal time had no influence on seroma formation [3]. The findings from our study also indicated that the length of time drains are left did not influence the seroma rate (Table 2). Similar observation was reported by a recent study where the use of drains did not prevent seroma formation. On the other  hand it was associated with a longer postoperative hospital stay and more pain after surgery for breast cancer [16]. It has been suggested that the restriction of arm movements may also reduce the incidence of seroma formation [8]. This observation however was challenged by others who showed that there is no significant disadvantage in early arm motion [9]. Porter *et al *reported that the use of electrocautery to create skin flaps in mastectomy reduces blood lose but increased the rate of seroma formation [11]. In addition, an association of postoperative seroma formation with neoadjuvant chemotherapy was also noted [4]. Compression dressing to prevent seroma rate is a common method used by many surgeons. A study demonstrated that routine use of a pressure garment to reduce postoperative drainage after axillary lymph node dissection for breast cancer is not warranted [12]. However, we think that the use of pressure garment and prolonged limitation of arm activity not only reduces seroma formation but also may increase the incidence of seroma formation after removal of drain [12] and even might cause shoulder dysfunction [8].

In the present study no relationship was observed between the incidence of seroma and the patients' age, tumor size, and lymph node status. However, the study found that the MRM was associated with higher rate of postoperative seroma formation (OR = 2.83, P = 0.04). Similarly, Gonzalez *et al*, demonstrated that patients who underwent modified radical mastectomy had a greater incidence of seroma formation than patients who underwent breast preservation surgery [10]. They also showed that there was a direct correlation between age and the development of seroma [10]. A recent study by Lumachi *et al *indicated that the tumor size and total amount of drainage represented the principal factors of seroma formation following axillary dissection in patients underwent surgery for breast cancer [19].

The results of our study suggest that seroma formation after breast cancer surgery is independent of duration of drainage, compression dressing and other known prognostic factors in breast cancer patients except the type of surgery, i.e there is a 2.5 times higher risk of seroma formation in patients undergoing MRM compared to BP. The small sample size of present study is a limitation and hence the power of the study is low. A number of questions remain unanswered and more research is needed to answer these.

## Conflict of interest

The authors declare that they have no competing interests.

## Source of Funding

None

## Contributors

**EH **designed the study, collected the data and wrote the first draft of the manuscript.

**AK, MN, ME **and **HH **all contributed to patient recruitment and the preparation of first draft of the manuscript.

**AM **contributed to the study design, data analysis, and edited the final version.

All authors read and approved of the manuscript.
